# Epigenetically downregulated Semaphorin 3E contributes to gastric cancer

**DOI:** 10.18632/oncotarget.3936

**Published:** 2015-05-12

**Authors:** Hui Chen, Guo-Hua Xie, Wei-Wei Wang, Xiang-Liang Yuan, Wen-Ming Xing, Hong-Jing Liu, Jin Chen, Min Dou, Li-Song Shen

**Affiliations:** ^1^ Department of Clinical Laboratory, Xinhua Hospital, Shanghai Jiao Tong University School of Medicine, Shanghai 200092, China; ^2^ Department of Academy, Shanghai Association for Science & Technology, Shanghai 200020, China

**Keywords:** Sema3E, gastric cancer, epigenetics, proliferation, migration

## Abstract

Axon guidance protein Semaphorin 3E (Sema3E) promotes tumor metastasis and suppresses tumor cell death. Here, we demonstrated that Sema3E was decreased in gastric cancer. Its levels were inversely associated with tumor progression. Levels of Sema3E were associated with low p300 and high class I histone deacetylase (class I HDAC). Ectopic expression of Sema3E inhibited proliferation and colony formation of gastric cancer cell lines *in vitro* and xenografts *in vivo*. Sema3E overexpression inhibited migration and invasion of gastric cancer cells, which was associated with induction of E-cadherin and reduction of Akt and ERK1/2 phosphorylation. We suggest that silencing of Sema3E contributes to the pathogenesis of gastric cancer.

## INTRODUCTION

Gastric cancer is the fourth most common cancer and the second leading cause of cancer mortality worldwide; it accounts for approximately 10% of cancer deaths according to GLOBOCAN 2012 estimates. The incidence and mortality of gastric cancer are the highest in East Asia, especially in South Korea, Mongolia, Japan and China.

As is the case with other cancers, gastric carcinogenesis is a multistep and multifactorial process that involves environmental factors, genetic alterations [1] and epigenetic alterations [2]. Apart from *H.pylori* infection, a major recognized risk factor for gastric cancer [3], smoking [4], diet and related nutrient intake [5], geographic, ethnic and cultural factors [6] among others, also play a vital role in the genesis and progression of gastric cancer.

Thus far, several susceptibility genes including TP53, KRAS, ARID1A, and ERBB3, among others, have been found to be related to gastric cancer [7]. In recent years, epigenetic mechanisms that govern gastric cancer, comprising DNA methylation, histone modification, microRNAs, and long non-coding RNAs, have become the focus of gastric cancer research [8]. It has also been shown that genes involved in cancer-related pathways are more frequently affected by epigenetic alterations than by genetic alterations [9]. Although an increasing number of studies have been conducted regarding the etiology of stomach cancer, the underlying mechanisms are not fully understood.

Semaphorins are a family of conserved membrane-associated proteins that are secreted and use plexin proteins as their primary receptors for signal-transduction. The deregulation of semaphorins and their receptors is frequently observed in cancers. Sema3E, a member of the semaphorin family, was initially found to act as a critical regulator in axon pathway guidance [10] and in vascular pattern formation [11].

Thus far, the role of Sema3E in several cancer types has been documented. The expression of Sema3E is positively associated with metastatic potential in breast cancer [12], ovarian cancer [13], melanoma cancer and colon cancer [14]. In colorectal cancer and pancreatic cancer, Sema3E expression is inversely correlated with tumor prognosis [14, 15]. We checked the expression of *SEMA3E* in databases of COSMIC, ICGC and TCGA that contain microarray or deep sequencing data using gastric cancer samples. *SEMA3E* missence mutation, stop gained mutation, frameshift, and low-level gain of the mutated *SEMA3E* affected a part of gastric cancer samples [7, 16, 17]. Therefore, Sema3E is likely to play a pivotal role in gastric carcinogenesis and progression, but this concept requires further study. Moreover, the mechanisms that lead to the abnormal expression of Sema3E in cancer have yet to be addressed.

In this study, we provide evidence of frequent down-regulation of Sema3E in gastric cancer. Abnormal expression of p300 and class I HDAC in gastric cancer may contribute to Sema3E silencing. Both *in vitro* and *in vivo* experiments demonstrated that Sema3E could inhibit the proliferation of gastric cancer cell lines, which was achieved by inhibition of entry into S phase during cell cycle progression and by promotion of apoptosis. Furthermore, Sema3E could suppress the migration and invasion of gastric cancer cells *in vitro*. ERK1/2, Akt and E-cadherin were involved in the downstream signaling of Sema3E.

## RESULTS

### Sema3E is frequently down-regulated in gastric cancer and inversely associated with tumor stage

**Figure 1:**
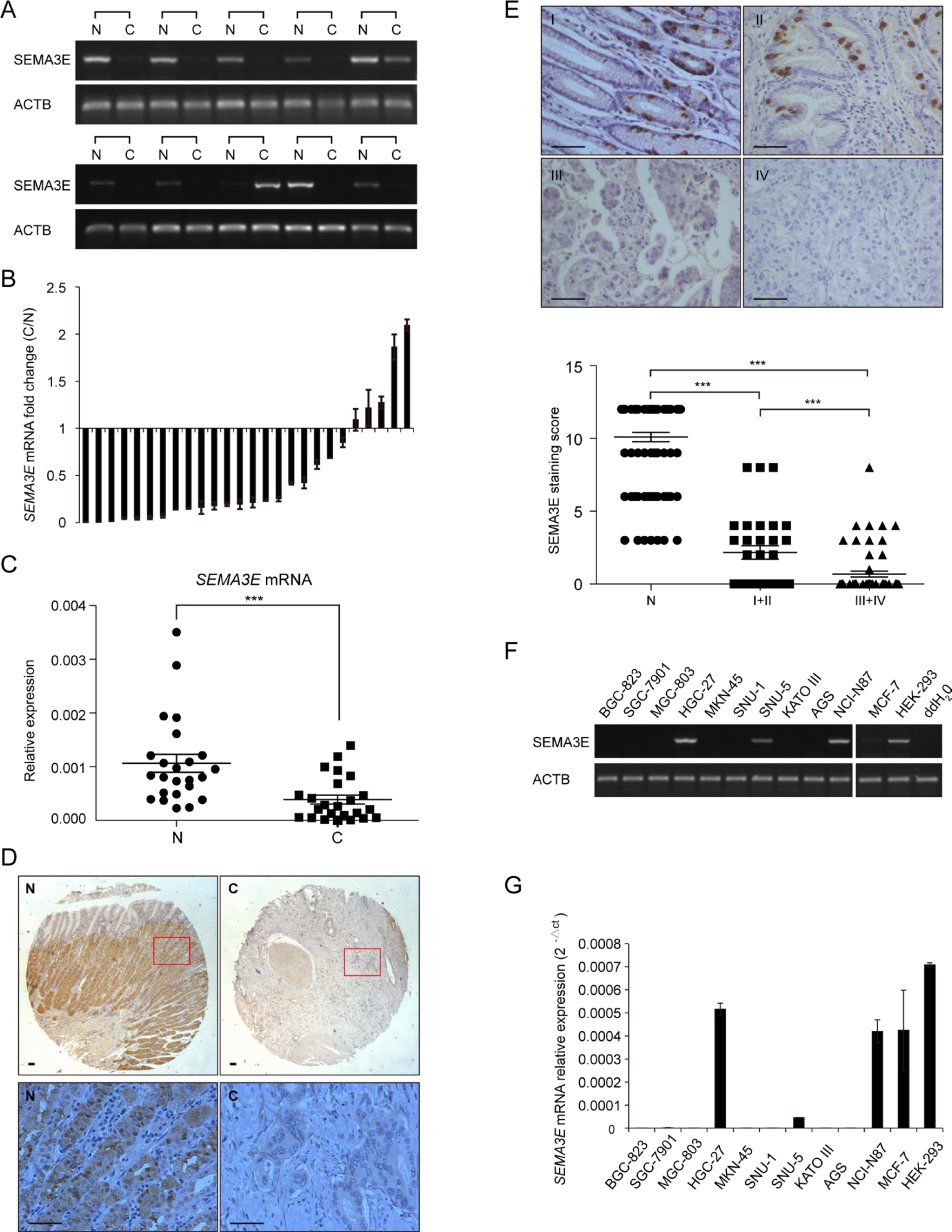
Sema3E is down-regulated in gastric cancer and gastric cancer cell lines **A.** RT-PCR indicates the expression of *SEMA3E* mRNA in 10 pairs of human gastric cancer and adjacent nontumor samples. **B.** Real-time PCR indicates the expression of *SEMA3E* mRNA in 26 pairs of human gastric cancer and corresponding noncancer samples. The expression level of *SEMA3E* was normalized to an endogenous control (*GAPDH*). **C.** Statistical analysis was performed by paired *t*-test to compare the expression level of *SEMA3E* presented in (B). **D.** Representative Sema3E immunohistochemistry on sections of paired gastric cancer and adjacent noncancer tissue is shown. The bottom panel is enlarged from the boxed region of the top image. Scale bar is 50 μm. **E.** Representative Sema3E immunohistochemistry on sections of TNM I to TNM IV gastric cancer is shown. Decreased Sema3E is correlated with the progression of gastric cancer. In TNM III and TNM IV stages of gastric cancer, Sema3E was barely detectable. Scale bar is 50 μm. **F and G.** The expression of *SEMA3E* was determined by RT-PCR and real-time PCR in gastric cancer cell lines, MCF-7 and HEK-293 cells. *ACTB* and *GAPDH* were used as endogenous controls respectively. The data are presented as the mean ± SD. ***P < 0.001 versus the control.

First, the expression of Sema3E was evaluated in gastric cancer. As shown in Fig. 1A, mRNA level of *SEMA3E* was significantly down-regulated in gastric cancer compared with corresponding adjacent normal tissues. Quantitative real-time PCR of *SEMA3E* in a panel of 26 pairs of tissues agreed with the initial PCR result, and obvious down-regulation of *SEMA3E* mRNA was observed in 21/26 (80.77%) gastric cancer tissues when the cut-off was set as 1(Fig. 1B and 1C, *P* < 0.001).

Immunohistochemical assay confirmed the substantially decreased expression of Sema3E in gastric cancer in 90/90 (100%) pairs of tissues (Fig 1D and S1A). In addition, the level of Sema3E decreased gradually with gastric cancer progression, and in TNM III and IV gastric cancer tissues Sema3E protein was barely detectable (Fig. 1E, *P* < 0.001). The association between Sema3E expression and the clinicopathological characteristics of the 90 patients with gastric cancer was analyzed and the results were summarized in Table 1. The expression level of Sema3E was significantly and inversely correlated with tumor volume (*P* < 0.05), lymphatic invasion (*P* < 0.05) and gastric cancer progression (*P* < 0.001).

**Table 1:** The correlation of Sema3E expression with clinicopathological features of gastric cancer

Clinicopathological feature	Number of cases	Expression of Sema3E (mean ± SEM)	*P* value
Gender			
Male	70	1.0290 ± 0.2416	0.2000
Female	20	1.7000 ± 0.4818	
			
Age (years)			
≥ 60	61	1.4430 ± 0.2952	0.0766
< 60	29	0.6207 ± 0.2350	
			
Tumor volume (cm^3^)			
≥ 45	32	0.5938 ± 0.2370	0.0345*
< 45	55	1.4360 ± 0.3122	
			
Lymphatic invasion			
≥ 8	23	0.5652 ± 0.2579	0.0321*
< 8	67	1.3880 ± 0.2740	
			
TNM stage			
I + II	30	2.1670 ± 0.4651	0.001***
III + IV	60	0.6833 ± 0.2024	

* *P* < 0.05,

*** *P* < 0.001.

To test Sema3E antibody for use in immunohistochemical analysis, we evaluated Sema3E expression in prostate cancer, mammary cancer, ovarian cancer and uterine cancer (Fig. S1B). Sema3E was determined to be increased in these tumors, which was in agreement with previous reports [12, 13, 18].

An analysis of *SEMA3E* expression in gastric cancer cell lines by PCR and quantitative real-time PCR showed that a majority of gastric cancer cell lines displayed low *SEMA3E* levels compared with HEK-293 and MCF-7 cells (Fig. 1F and 1G), two cell lines reported to express *SEMA3E*. Moreover, no difference was observed between gastric cancer and adjacent normal tissues with respect to *PLXND1*mRNA, the Sema3E-specific receptor (Fig. S2A and S2B).

These results suggest that down-regulation of Sema3E in gastric cancer correlates with gastric cancer progression.

### P300 and class I HDAC are involved in the down-regulation of Sema3E in gastric cancer

**Figure 2:**
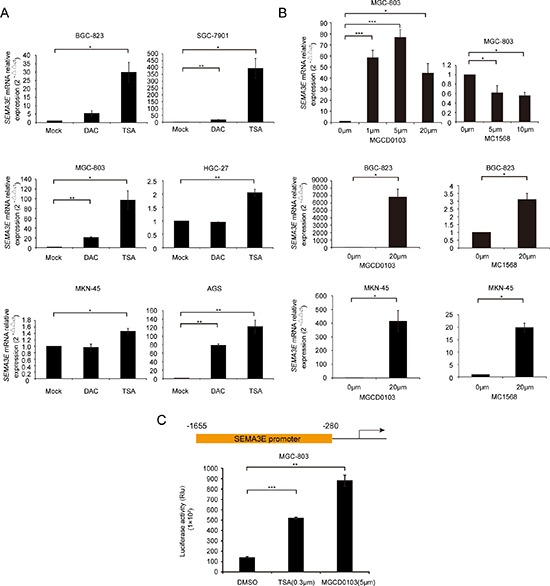
Epigenetic regulatory mechanisms are involved in SEMA3E transcription **A.** The re-expression of *SEMA3E* in gastric cancer cell lines treated with DMSO, DAC or TSA was evaluated by real-time PCR. *GAPDH* was used as endogenous control. **B.** The re-expression of *SEMA3E* in gastric cancer cell lines treated with DMSO, MGCD0103 or MC1568 was evaluated by real-time PCR. *GAPDH* was used as endogenous control. Statistical analysis was conducted with paired *t*-test. **C.** The −1655 bp to −280 bp sequence upstream of *SEMA3E* transcription initiation site was likely *SEMA3E’*s promoter, as predicted by GeneCopoeia. Luciferase activity of *SEMA3E* promoter in MGC-803 cells treated with TSA or MGCD0103 was assessed with Luciferase Assay System. DMSO treatment was used as control. The data are presented as the mean ± SD. ***P < 0.001, **P < 0.01, *P < 0.05 versus the control.

To identify whether epigenetic mechanisms might contribute to the deregulation of Sema3E in gastric cancer, 6 gastric cancer cell lines were treated with DNA demethylating agent 5-aza-2′-deoxycytidine (DAC) or pan histone deacetylase inhibitor trichostatin A (TSA). *SEMA3E* mRNA levels increased slightly or remained unchanged after DAC treatment (Fig. 2A), which is consistent with the fact that no CpG island was found within *SEMA3E* promoter (Fig. S2C). The reason that DAC can slightly increase *SEMA3E* expression in SGC-7901, MGC-803 and AGS maybe in these cell lines DAC promoted other genes then increased *SEMA3E* expression. In contrast, TSA strongly induced *SEMA3E* expression that *SEMA3E* mRNA was markedly up-regulated up to 100 times that of the baseline in BGC-823, SGC-7901, MGC-803 and AGS after TSA treatment (Fig. 2A). To determine which class of HDACs functions in the transcription of *SEMA3E*, three gastric cancer cell lines were treated with the following class-specific HDAC inhibitors: class I HDAC inhibitor mocetinostat (MGCD0103) or class II HDAC inhibitor MC1568. MGCD0103 significantly enhanced *SEMA3E* levels over a dozen-fold, up to 100-fold and up to 1000-fold in MGC-803, MKN-45 and BGC-823, respectively. However, MC1568 did not exert such significant effect (Fig. 2B). Furthermore, luciferase reporter assay demonstrated that in MGC-803 cells, TSA as well as MGCD0103 markedly induced the activity of the *SEMA3E* promoter (Fig. 2C). As Fig. 2B showed 5 μm was the best concentration of MGCD0103 to induce the expression of *SEMA3E* in MGC-803 cell line, so 5 μm MGCD0103 was used to induce the activity of *SEMA3E* promoter. Meanwhile, MC1568 can induce the activity of *SEMA3E* promoter only a little (Fig. S2D).

**Figure 3:**
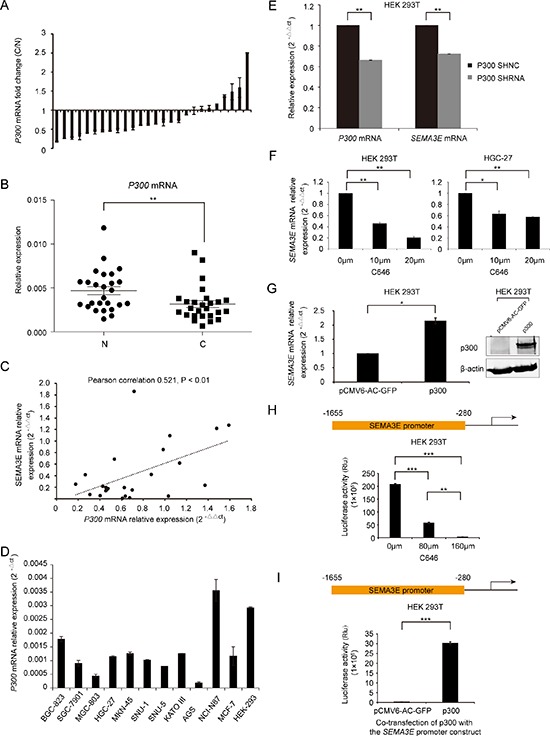
The histone acetylation enzyme p300 plays a role in the regulation of *SEMA3E* transcription **A.** Real-time PCR indicates the expression of P300 mRNA in 26 pairs of human gastric cancer and corresponding noncancer samples. The expression level of P300 was normalized to an endogenous control (GAPDH). **B.** Statistical analysis was performed by *t*-test to compare the level of P300 presented in (A). **C.** The correlation of P300 and SEMA3E in 24 pairs of gastric cancer and corresponding adjacent normal tissues. **D.** The expression of P300 mRNA in gastric cancer cell lines, MCF-7 and HEK-293 cells was determined by real-time PCR. GAPDH was used as endogenous control. **E.** The expression level of SEMA3E after p300 knockdown in HEK 293T was determined by real-time PCR and normalized to GAPDH. Statistical analysis was performed by paired *t*-test. **F.** The expression level of SEMA3E in HEK 293T and HGC-27 cells treated with C646 was determined by real-time PCR and normalized to GAPDH. **G.** The level of SEMA3E in HEK 293T with p300 overexpression was determined by real-time PCR and normalized to GAPDH. **H.** Luciferase activity of SEMA3E promoter in HEK 293T cells that were treated with C646. **I.** Luciferase activity of *SEMA3E* promoter in HEK 293T co-transfected with p300 was analyzed. Discrepancies in the transfection efficiency between the GFP and the p300 group were eliminated by sorting the same number of transfection-positive cells with BD FACS CANTO II flow cytometer cell sorting system (BD Biosciences, USA) according to GFP tag. Statistical analysis was performed by paired *t*-test. The data are presented as the mean ± SD. ***P < 0.001, **P < 0.01, *P < 0.05 versus the control.

The histone acetyltransferase p300, a potential tumor suppressor in gastric cancer [19], has been reported to be down-regulated in gastric cancer. We validated that *P300* was reduced in the 26 pairs of gastric cancer tissues (Fig. 3A and 3B, *P* < 0.01). And *P300* expression correlated positively with *SEMA3E* expression in 24 pairs of tissues (*P* < 0.01) (Fig. 3C). Results of two pairs of samples with highest expression of *P300* or *SEMA3E* were excluded from the correlation analysis. *P300* expression in gastric cancer cell lines was, on average, low (Fig. 3D). Interference of p300 expression inhibited the transcription of *SEMA3E* (Fig. 3E). Additionally, C646, a specific p300 histone acetylase (HAT) inhibitor, attenuated *SEMA3E* expression in HEK 293T and HGC-27 cells (Fig. 3F). Meanwhile, ectopic expression of p300 significantly induced the expression of *SEMA3E* in HEK 293T cell (Fig. 3G). Moreover, *SEMA3E* promoter exhibited a dose-dependent repression (72.4% to 98.5%) according to luciferase activity in HEK 293T cell when treated with 80 μm C646 and 160 μm C646, respectively (Fig. 3H). On the contrary, co-transfection of p300 with *SEMA3E* promoter construct led to more than 100-fold augmentation in luciferase activity compared with the control (Fig. 3I).

These results imply that p300 and class I HDAC have an active role in the transcription and expression of Sema3E in gastric cancer.

### Overexpression of Sema3E restricts the proliferation and adhesion-dependent colony formation in gastric cancer cell lines *in vitro*

**Figure 4:**
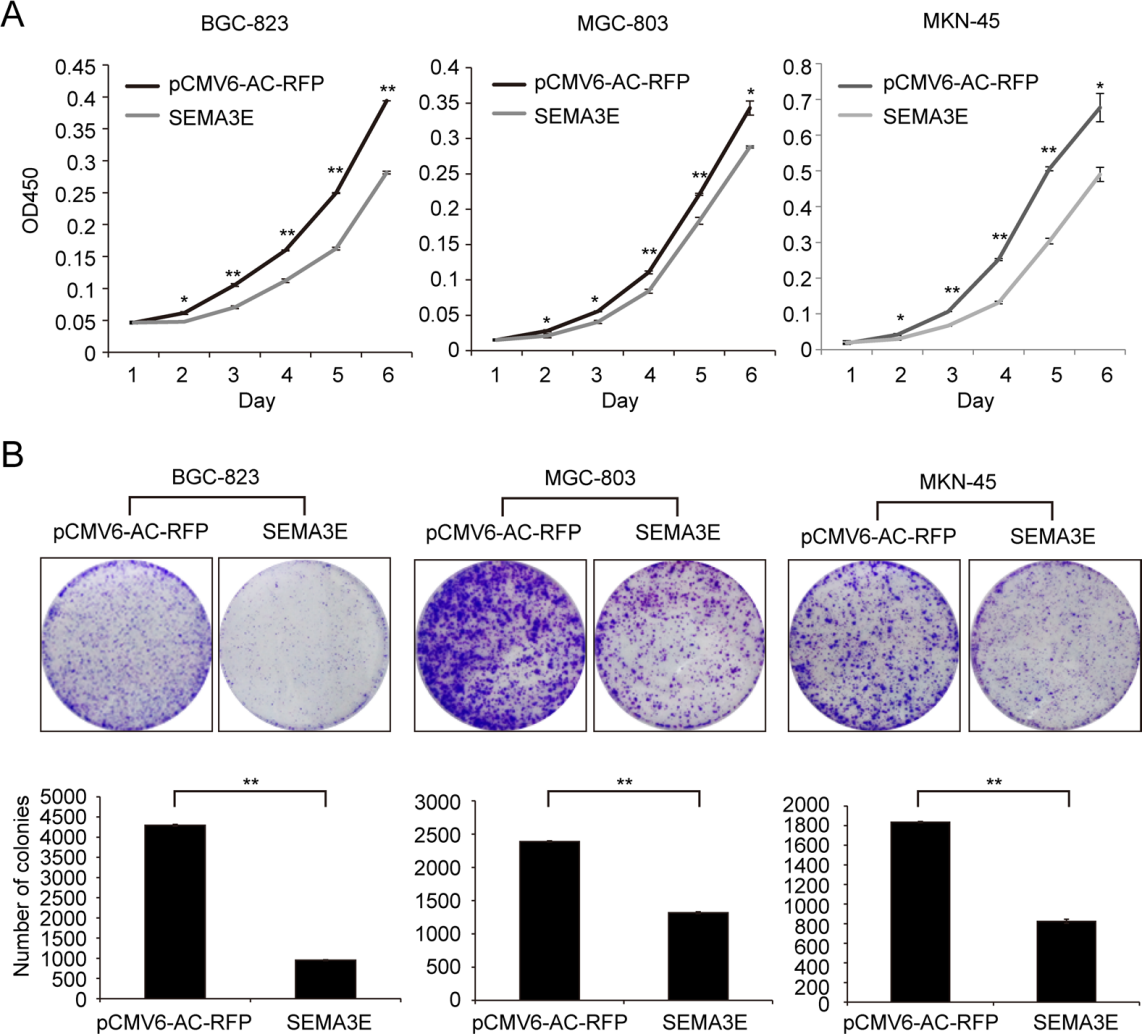
Sema3E inhibited proliferation and colony formation of gastric cancer cells *in vitro* **A.** Forced Sema3E expression attenuated the proliferation of the three cell lines. **B.** Ectopic Sema3E expression inhibited colony formation of the three gastric cancer cell lines. Representative colony formation pictures of cells transfected with *SEMA3E* and control vector are shown. The histograms represent the number of colonies, and the data are shown as the mean ± SD. The experiments were all repeated at least 3 times to confirm the reproducibility of the results. **P < 0.01, *P < 0.05 versus the control.

To examine the effect of Sema3E on gastric cancer, recombinant plasmid SEMA3E-pCMV6-AC- RFP was transiently transfected into 3 gastric cancer cell lines with low baseline Sema3E expression (BGC-823, MGC-803 and MKN-45). This approach was based on the results of *SEMA3E* expression profile in gastric cancer cell lines shown in Fig. 1F and 1G. Fluorescence microscopy and western blot were then performed to evaluate the transfection efficiency of the *SEMA3E* recombinant plasmid (Fig. S2E and S2F). The viability of the transfected cell lines was monitored for 6 consecutive days. The results indicated that Sema3E could appreciably suppress the proliferation of gastric cancer cells (Fig. 4A). In addition, adhesion-dependent colony formation assay further validated the ability of Sema3E to inhibit the proliferation of gastric cancer cells, as ectopic *SEMA3E*-transfected gastric cancer cells displayed a decrease in the number of colonies and smaller colony size (Fig. 4B). The opposite effect can be achieved by knockdown of Sema3E in HGC-27 cell line with Sema3E ShRNA1 and ShRNA2 (Fig. S3B and S3C). Knockdown effect of Sema3E can be found in Fig. S3A. These data suggest that Sema3E exerts a tumor suppressive effect on gastric cancer cell lines.

### Sema3E inhibits cell proliferation by blocking entry into S phase during cell cycle progression and promotion of apoptosis

**Figure 5:**
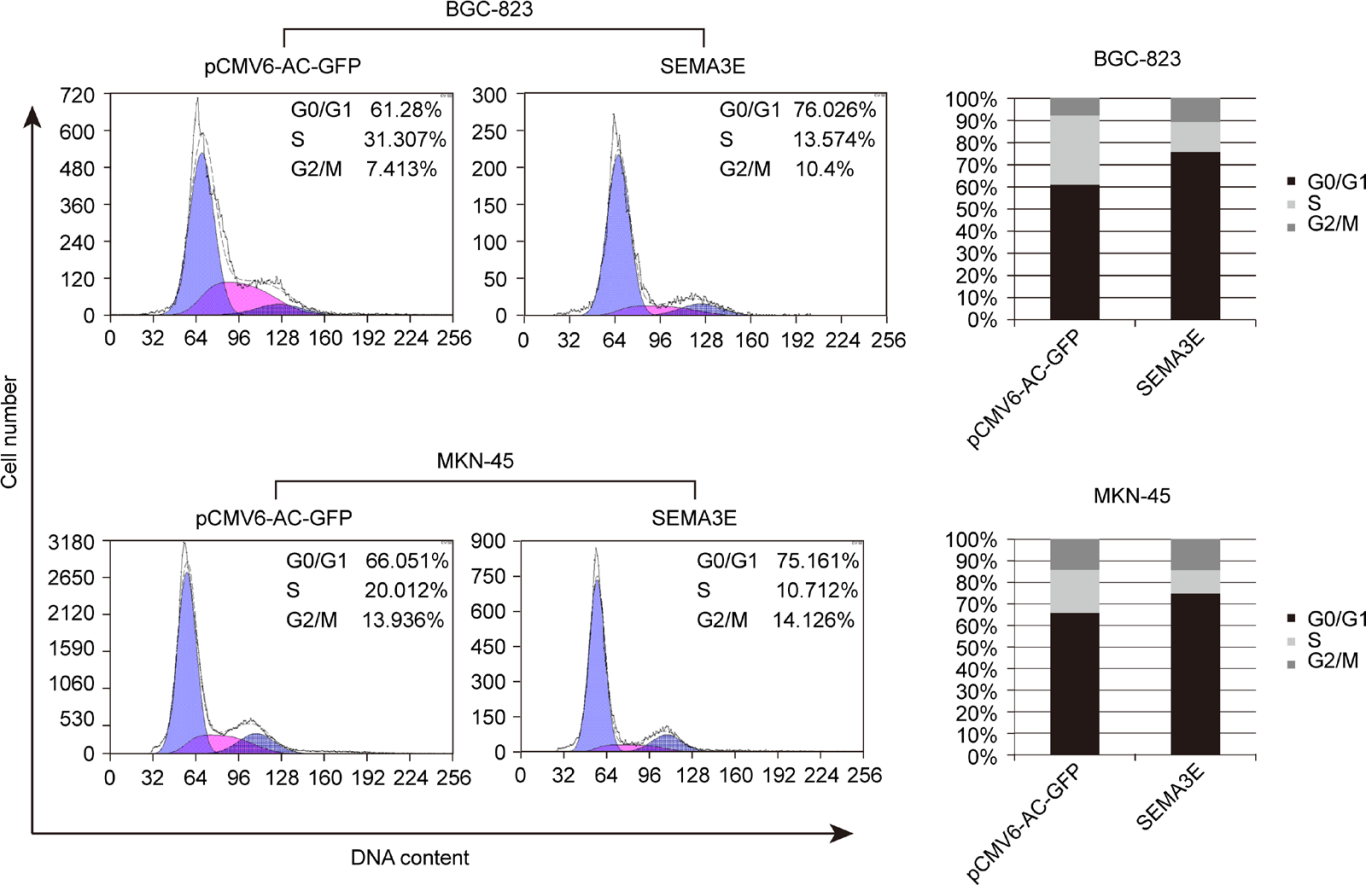
Sema3E delayed G1-S transition during cell cycle progression in gastric cancer cells The cell cycle distribution was analyzed in BGC-823 and MKN-45 cells transfected with *SEMA3E* or empty vector. The histograms represent the means.

**Figure 6:**
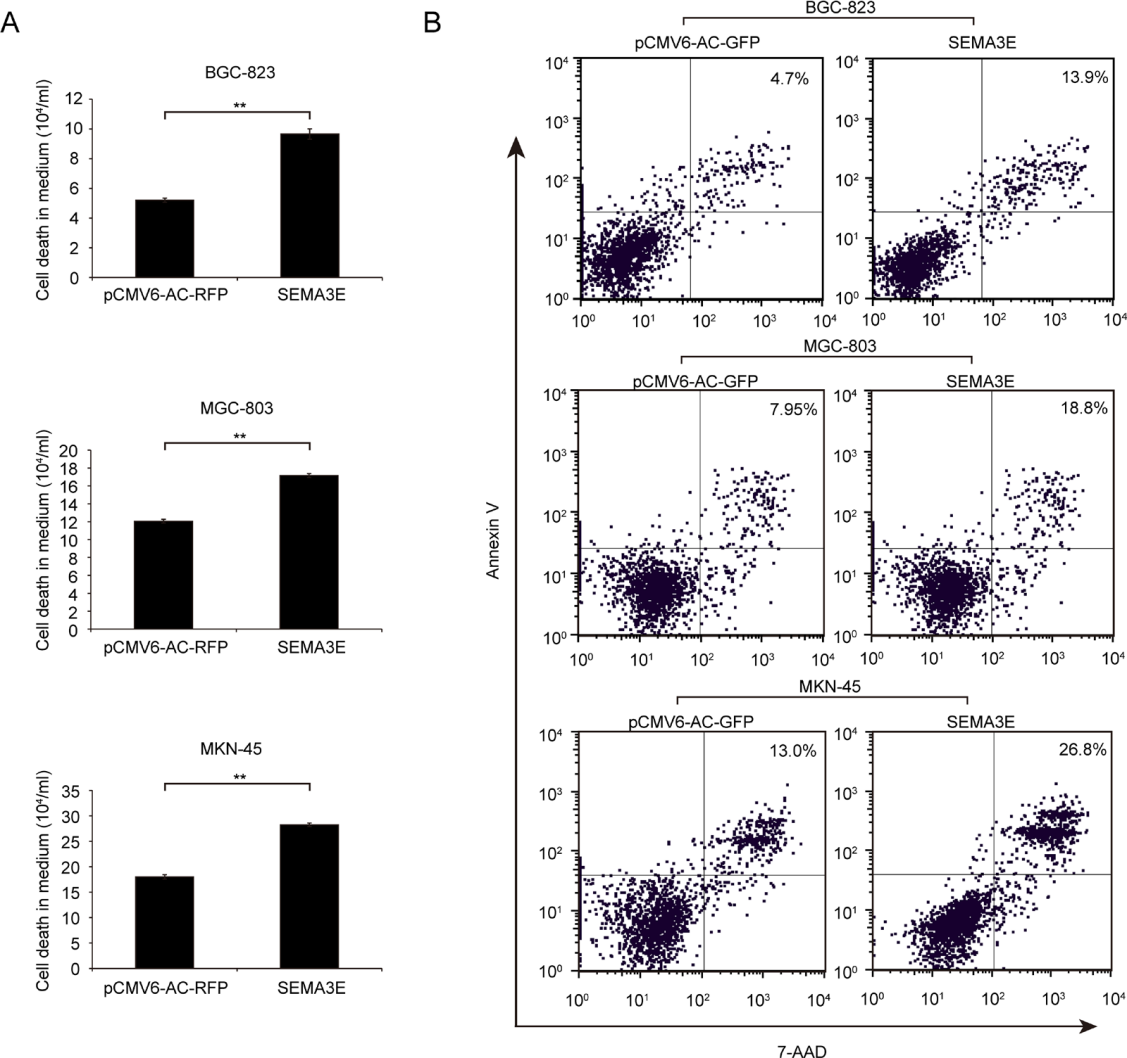
Sema3E promoted apoptosis of gastric cancer cell lines **A.** Trypan blue staining was used to assess the number of dead cells in the cell culture supernatant of gastric cancer cell lines transfected with *SEMA3E* or with control vector. The histogram column indicates the number of dead cells, and the data are shown as the mean ± SD. Statistical analysis was conducted with paired *t*-test. **B.** The apoptosis of BGC-823, MGC-803 and MKN-45 cells transfected with *SEMA3E* or control vector was analyzed by Annexin V : PE Apoptosis Detection Kit. The percentage of cells in the late stage of apoptosis is shown. **P < 0.01 versus the control.

To assess the role of Sema3E in cell cycle progression and cell apoptosis, which are two leading causes of change in cell proliferation, we performed flow cytometric analysis. S phase cell fractions were significantly reduced in *SEMA3E*-transfected BGC-823 and MKN-45 gastric cancer cell lines compared with the control; correspondingly, the G0/G1phase cell fractions increased (Fig. 5). Trypan blue staining showed that the number of dead cells significantly increased in the cell culture supernatant of *SEMA3E*-transfected cells compared with control (Fig. 6A). An analysis of apoptosis with Annexin V : PE Apoptosis Detection Kit indicated that Sema3E enhanced late apoptosis of BGC-823, MGC-803 and MKN-45 (Fig. 6B). These data provide evidence to support the role of Sema3E in inhibition of gastric cancer cell proliferation by blocking the transition of cells in G0/G1 into S phase during cell cycle progression and through promotion of apoptosis.

### Overexpression of Sema3E attenuates the migration and invasion of gastric cancer cell line

**Figure 7:**
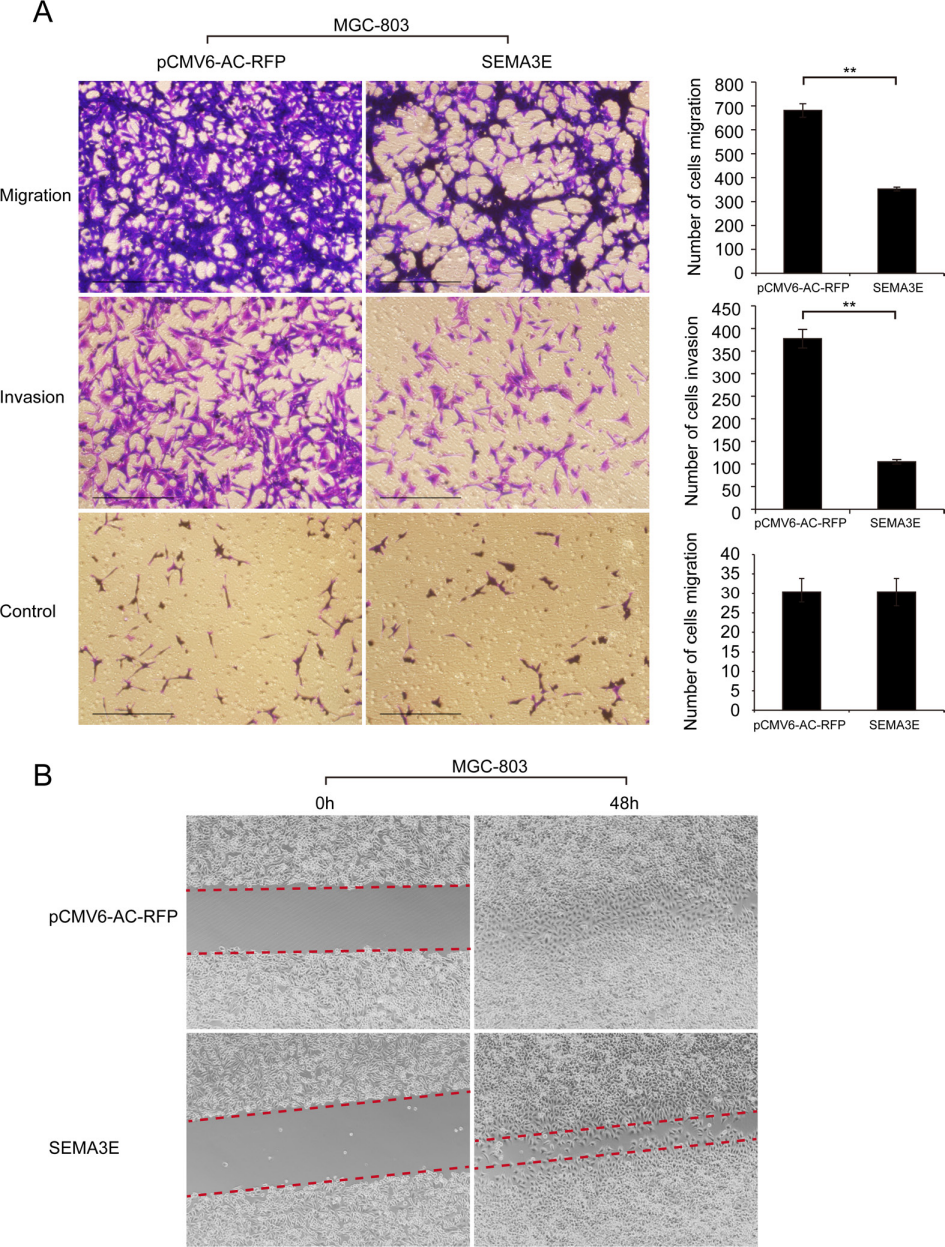
Sema3E modulated the migration and invasion of gastric cancer cell lines **A.** The migration and invasion abilities of MGC-803 gastric cancer cell line after transfection with SEMA3E were evaluated by BD migration and matrigel invasion assays. All experiments were repeated at least three times. Representative fields of cells that invaded are shown. The histogram indicates the number of cells that invaded; the data are shown as the mean ± SD. Scale bar is 500 μm. **B.** Wound healing experiment was used to analyze the migration of MGC-803 cells transfected with SEMA3E. **P < 0.01 versus the control.

To investigate whether Sema3E participates in gastric cancer migration and invasion *in vitro*, we employed transwells without or with matrigel to assess the effects of Sema3E on cell migration and invasion respectively. *SEMA3E* recombinant plasmid was transiently transfected into MGC-803 cells to evaluate these effects. Compared with cells that were transfected with empty vector, Sema3E overexpression significantly inhibited cell migration as well as cell invasion (Fig. 7A). Wound healing assay subsequently confirmed that Sema3E could restrain the migration of MGC-803 cells (Fig. 7B). However, Sema3E overexpression had no effect on the depolymerization of F-actin in MGC-803 cells according to phalloidin staining (Fig. S4A). Although it has been reported that recombinant Sema3E protein affected the depolymerization of F-actin in human airway smooth muscle cells [20]. Altogether, these data suggest that Sema3E inhibits the migration and invasion of MGC-803 cells.

### Sema3E reduces tumorigenicity and tumor burden in xenograft mouse model *in vivo*

**Figure 8:**
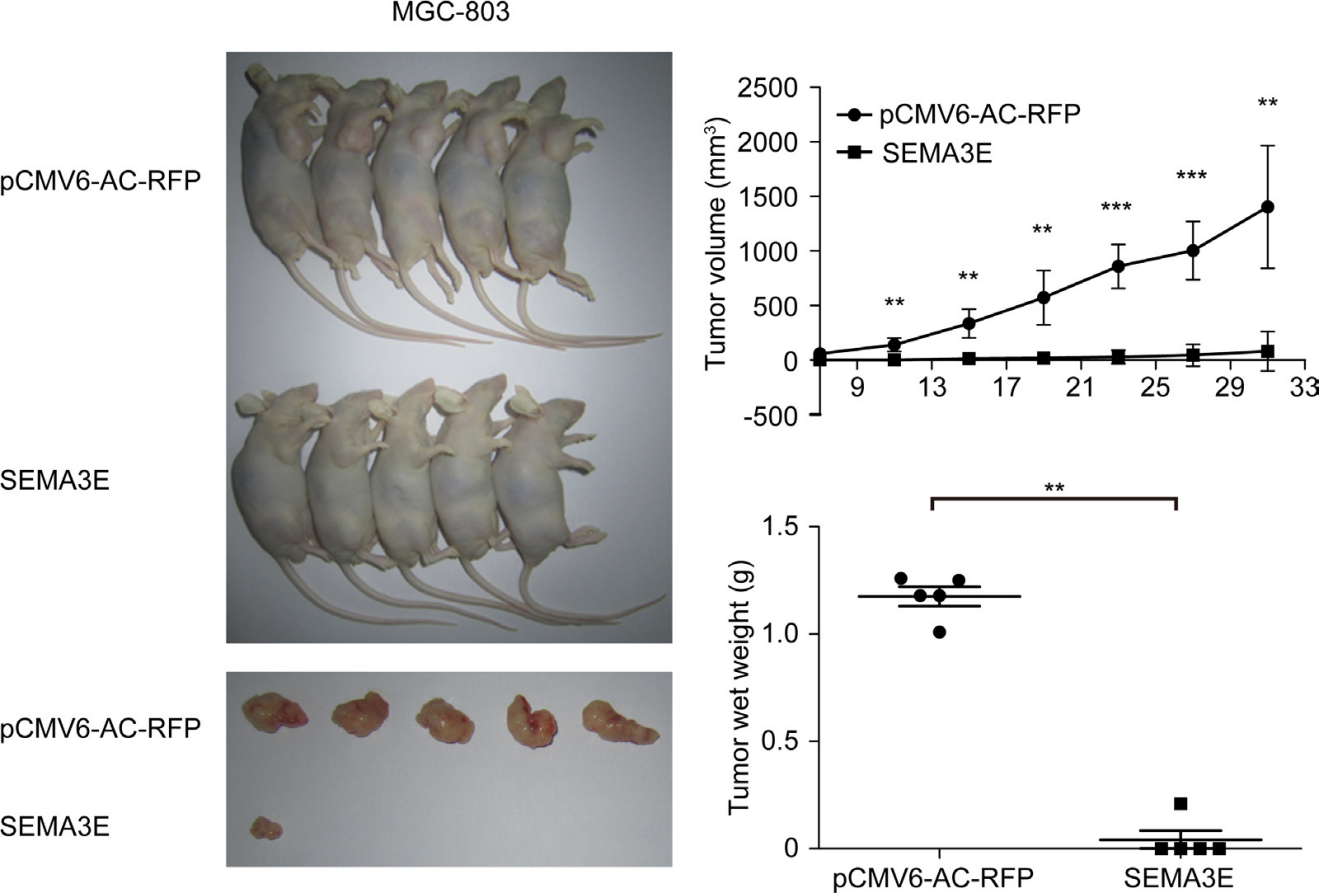
Sema3E lowered tumorigenicity of gastric cancer cell lines *in vivo* Sema3E inhibited tumorigenicity of MGC-803 cells *in vivo*. Cells that carried empty vector were used as negative control (*n* = 5). Tumor growth was monitored every 4 days by measuring the diameter. All the xenograft tumors were removed from the experimental mice and weighed (mean ± SD). ***P < 0.001, **P < 0.01 versus the control.

To investigate the effect of Sema3E on tumorigenicity *in vivo*, *SEMA3E* and control vector were transfected into MGC-803 gastric cancer cells. Transfection positive cells were then sorted by flow cytometry (BD FACSCANTO II, BD Biosciences, USA) according to RFP tag. Subsequently, these cells were inoculated into right armpit of nude mice. As expected, Sema3E reduced the occurrence of visible tumors from MGC-803 cells, and only 1 in 5 mice developed tumors from these cells. In contrast, the cells that were transfected with control vector produced tumors in all 5 mice. Sema3E significantly suppressed tumorigenicity of MGC-803 cells *in vivo*, and the size and weight of Sema3E-overexpressing tumors were clearly lower than those of xenografts formed from cells transfected with control vector (Fig. 8).

### Sema3E exerts an inhibitory effect on the phosphorylation of Akt and ERK1/2 and induces E-cadherin expression

**Figure 9:**
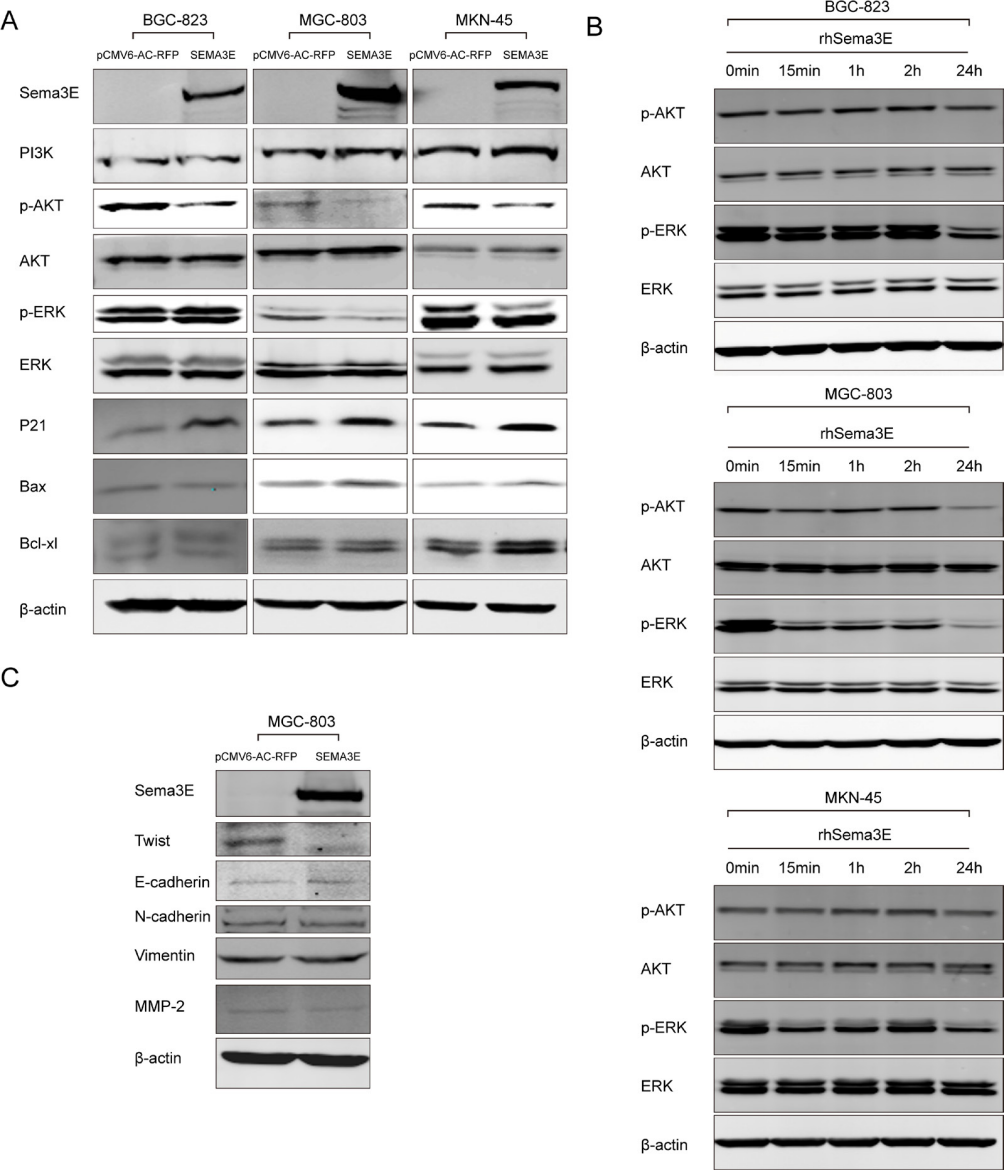
Sema3E inhibited ERK1/2 and Akt signaling and up-regulated E-cadherin via Twist reduction **A.** Canonical signaling pathway nodes were screened by western blot in BGC-823, MGC-803 and MKN-45 transfected with *SEMA3E*. **B.** Recombinant human Sema3E added in the culture medium could also pose inhibitory effect on ERK1/2 phosphorylation in the three gastric cancer cell lines and Akt phosphorylation in MGC-803. **C.** In MGC-803 cells with ectopic expression of Sema3E, signaling pathways involved in cell migration and invasion were screened.

To elucidate the signaling pathways that lead to the inhibitory effects of Sema3E on gastric cancer cells, western blot was performed to analyze the effect of Sema3E on several signaling pathways associated with cell proliferation, migration and/or invasion. Sema3E overexpression alone significantly reduced the phosphorylation of Akt and ERK1/2 (Fig. 9A). On the contrary, ERK1/2 phosphorylation increased after Sema3E knockdown (Fig. S3D). After recombinant human Sema3E was added to the culture media of BGC-823, MGC-803 and MKN-45 cell lines, similar effect with Sema3E overexpression was observed (Fig. 9B). However, in MKN-45 and BGC-823 Akt phosphorylation didn't change obviously. Maybe intracellular Sema3E played some different role from secreted form. P21 expression, which was increased by Sema3E, may be responsible for the inhibition of cells in G0/G1 phase to transit into S phase (Fig. 9A). Additionally, Sema3E likely enhanced apoptosis of gastric cancer cell lines via induction of Bax and Bcl-xl (Fig. 9A). Other signaling pathways did not reveal any change (Fig. S5A).

Twist was reduced by overexpression of Sema3E in MGC-803, while E-cadherin expression increased (Fig. 9C).

Our results showed that overexpression of MAPK3 (ERK1) can abolish Sema3E's inhibitory effect on MGC-803 migration. And overexpression of Twist, Akt and MAPK3 can abolish Sema3E's inhibitory effect on MGC-803 invasion (Fig. S4B). These data suggest that Twist/E-cadherin, ERK1/2, Akt are involved in Sema3E-mediated inhibition of gastric cancer cell migration and invasion.

### The full-length isoform of Sema3E exerts an inhibitory effect on gastric cancer cell lines

**Figure 10:**
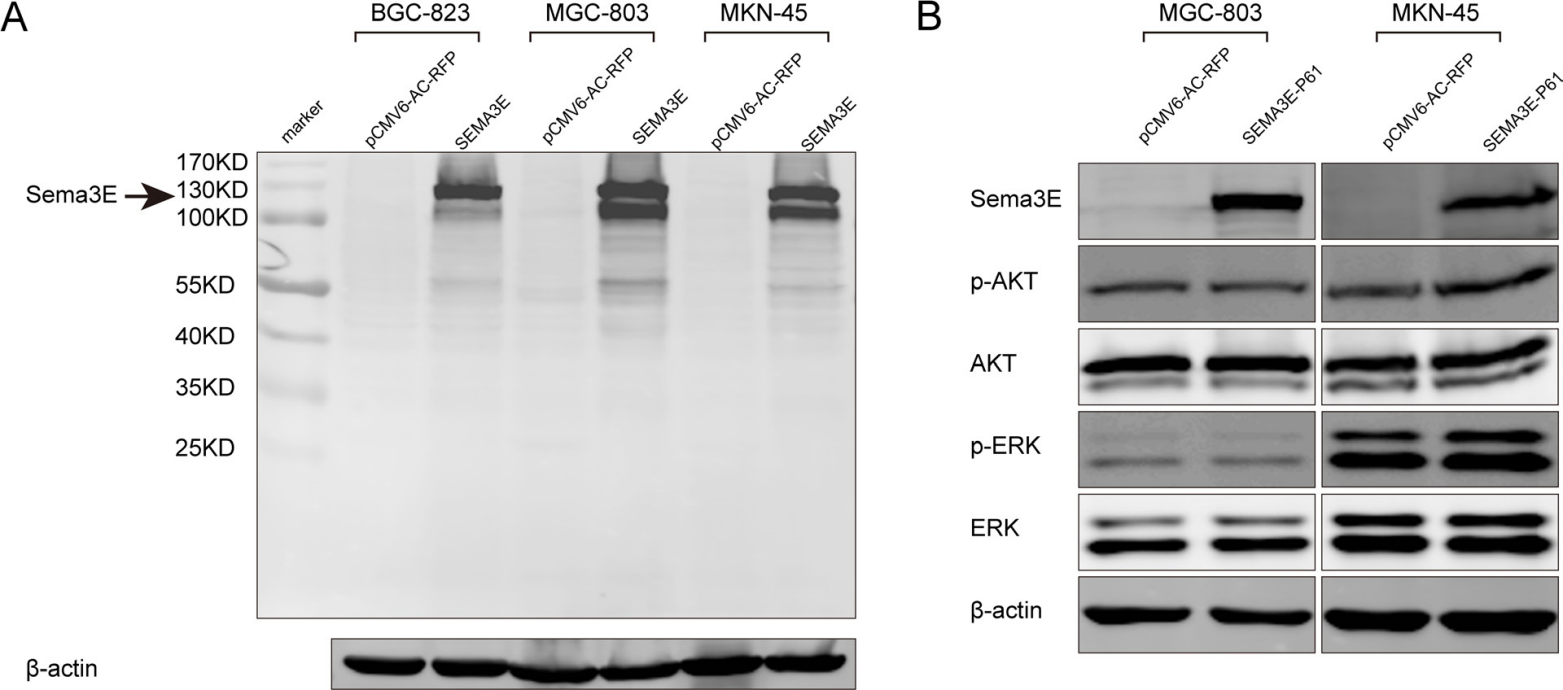
The full-length Sema3E protein exerted an inhibitory effect on gastric cancer cell lines **A.** Immunoblotting was used to assess the isoforms of Sema3E in gastric cancer cells transfected with *SEMA3E*. **B.** The P61 form of Sema3E was unable to inhibit ERK1/2 and Akt phosphorylation in gastric cancer cells.

Sema3E exists in several different isoforms, and furin is responsible for the proteolytic cleavage of Sema3E [21]. PCR result confirmed the high expression of *FURIN* in gastric cancer cell lines (Fig. S5B). However, no P61-Sema3E protein band was observed by western blot in gastric cancer cells (Fig. 10A). Meanwhile, ectopic expression of P61-SEMA3E construct could not reduce ERK1/2 and Akt phosphorylation (Fig. 10B). Together, the present evidence leads us to propose that it is the full-length Sema3E protein that participates in the inhibition of gastric cancer.

## DISCUSSION

The elucidation of molecular mechanisms that underlie the occurrence, progression and metastasis of gastric cancer is of vital importance, and will provide new clues with regards to treatment strategies for gastric cancer.

Sema3E/Plexin D1 signaling has been shown to have multi-faceted effects. The inhibition effect of cell proliferation and migration by Sema3E has been reported. For example, as a repulsive cue for Plexin D1-expressing endothelial cells [11], overexpression of Sema3E inhibits tumor development via the disruption of tumor vascular patterning [13, 14, 22, 23]; similarly, recombinant Sema3E can inhibit PDGF-mediated proliferation and migration of human airway smooth muscle cells [20]. In advanced atherosclerotic plaques, Sema3E is up-regulated, which may promote macrophage retention and chronic inflammation *in vivo* through negative regulation of macrophage migration [24]. Nevertheless, no report has been published on the inhibitory effect of Sema3E on the proliferation, migration or invasion of tumor cells until now. On the contrary, in some cancers, up-regulation of Sema3E promotes tumor metastasis. For instance, a 61KD isoform of Sema3E that is obtained by proteolytic processing by furin increases the invasiveness and distant metastasis of tumor cells [13, 14, 21, 25]. Sema3E has also been shown to suppress tumor cell death in metastatic breast cancer [12]. The expression and function of Sema3E in gastric cancer are currently unknown, and the mechanism that leads to the abnormal expression of Sema3E in cancers has not yet been documented.

Our study is the first to describe the expression of Sema3E as well as the epigenetic mechanism involved in the deregulation of Sema3E in gastric cancer and the functional consequences of Sema3E overexpression on the proliferation, migration and invasiveness of gastric cancer cell lines. We found that Sema3E was significantly decreased in gastric cancer. Down-regulation of p300 and up-regulation of class I HDAC may contribute to the decrease of Sema3E. Sema3E overexpression alone restricted the proliferation of gastric cancer cell lines, which may be caused by a delayed entry into S phase during cell cycle progression; it may also be caused by the promotion of apoptosis as well as a decrease in ERK1/2 and Akt phosphorylation. Otherwise, we found that Sema3E inhibited the migration and invasion of gastric cancer cells, which were associated with up-regulation of E-Cadherin and reduction of ERK1/2 and Akt phosphorylation.

As important forms of epigenetic regulation, histone acetylation and histone deacetylation have been demonstrated to be elaborately controlled by a highly specific regulatory process that leads to the activation and inactivation of certain genes. Ongoing activities of histone acetyltransferases (HATs) and histone deacetylases (HDACs) maintain the acetylation status of a specific lysine in a particular histone at a steady level. Histone hyperacetylation generally promotes gene transcription, while histone hypoacetylation usually represses gene expression [26].

Histone deacetylases (HDACs), a family of 18 molecules that are grouped into 4 classes, are enzymes that function in chromatin remodeling, gene expression and modification of histone and nonhistone proteins [27, 28]. Class I HDAC includes four members (HDAC1, 2, 3 and 8), which displays high enzyme activity toward histone substrates [28, 29]. Biochemical and genetic evidence have shown that class I HDAC is involved in various pathological conditions including cancer and it acts through modulation of histone acetylation. The abnormal expression of HDACs has been found in gastric cancer. Moreover, HDAC1 and HDAC2 have been reported to be up-regulated in gastric cancer [30–33]. Our work has revealed that class I HDAC regulated *SEMA3E* transcription and may be involved in Sema3E down-regulation in gastric cancer; however, as for which HDAC is responsible for these effects requires further investigation.

The homologous nuclear proteins p300 and CBP are transcriptional co-activators that they can couple with various transcription factors to act as a protein scaffold in order to facilitate gene expression [34]. Moreover, p300 can acetylate histones through its inner histone acetyltransferase (HAT) activity, which results in chromatin remodeling and the accessibility of transcription factors to DNA templates; this activity, in turn, promotes gene transcription [35, 36]. Accumulating evidence has connected p300 with cancer. Somatic missense mutations coupled to the deletion of the second allele of p300 were detected in gastric cancer, which yielded important insight into p300 as a tumor suppressor in gastric cancer [19].

As both p300 and Sema3E are inactivated in gastric cancer and p300 can promote gene transcription through its HAT activity, we sought to find a possible relationship between these two proteins. Fortunately, we were able to demonstrate a positive correlation between Sema3E and p300 in gastric cancer. Additionally, both knockdown of p300 and inhibition of the HAT activity of p300 with C646 were able to suppress *SEMA3E* transcription, while ectopic expression of p300 induced Sema3E expression. Moreover, luciferase reporter assay confirmed that p300 was involved in *SEMA3E* transcription.

Both ERK1/2 and Akt are important signaling pathways that regulate cell growth, differentiation, migration and invasion [37–42]. Sema3E was found to inhibit the phosphorylation of ERK1/2 and Akt in gastric cancer cell lines, which resulted in the inhibition of proliferation, migration and invasion.

Down-regulation of E-cadherin plays an essential role in epithelial-mesenchymal transition (EMT) during the early steps of invasion and metastasis. Transcription factor Twist was demonstrated to be activated and induce EMT in the process of tumor metastasis through inhibition of E-cadherin transcription [43]. Our study showed that ectopic expression of Sema3E inhibited Twist expression and increased E-cadherin expression. Twist overexpression abolished Sema3E's inhibitory effect on gastric cancer cells invasion. Together, the present evidence leads us to propose that up-regulation of E-cadherin and reverse of EMT contribute to Sema3E's inhibitory effect on gastric cancer cells invasion.

In conclusion, our study provides evidence that silencing of Sema3E contributes to gastric cancer progression and metastasis. As a novel finding, down-regulation of Sema3E in gastric cancer may result from reduction of p300 and up-regulation of class I HDAC. Our results give new insight into the control of gastric cancer progression.

## MATERIALS AND METHODS

### Clinical samples

All gastric cancer specimens were obtained from patients who underwent surgical resection. Informed consent was obtained before the surgeries were performed at Xinhua Hospital, Shanghai Jiao Tong University School of Medicine (Shanghai, China) between 2007 and 2008. Both primary tumor and adjacent nontumor tissues were collected from each patient and validated by pathological examination. Ninety paired gastric cancer tissues and adjacent normal tissues were prepared as part of tissue array for immunohistochemistry, while 26 paired tissues were frozen immediately after collection for RNA extraction. The use of human specimens and all of the experimental procedures in this study were approved by the ethics committee of Xinhua Hospital.

### Immunohistochemical (IHC) staining

Standard immunohistochemical procedures were performed on prepared five-micrometer thick paraffin-embedded tumor and adjacent nontumor sections with an anti-Sema3E polyclonal antibody (LifeSpan BioSciences, Inc., USA). Normal rabbit IgG was used in place of the primary antibody as negative control. The staining intensity (0, no staining; 1, weak; 2, moderate; and 3, intense) and the proportion of stained cells (0, no staining; 1, < 10%; 2, 11%–33%; 3, 34%–66%; and 4, >67%) were semi-quantitatively determined. The immunoreactive score was obtained by the sum of these 2 scores (negative, 0; weak, 1–4; moderate, 5–8; strong, 9–12).

### Cell lines and culture conditions

Ten gastric cancer cell lines (BGC-823, SGC-7901, MGC-803, HGC-27, MKN-45, SNU-1, SNU-5, KATO III, AGS, NCI-N87) derived from gastric tumors, one breast cancer cell line (MCF-7) derived from mammary tumor and two cell lines derived from fetal kidney (HEK-293 [44], HEK 293T [45]) were used in this investigation. These cell lines were cultured in Dulbecco's Modified Eagle Media (DMEM, Hyclone, USA) supplemented with 10% Fetal Bovine Serum (FBS, Gibco, USA), 100 U/mL penicillin and 100 μg/mL streptomycin. All of these cell lines were maintained at 37°C in a humidified incubator with 5% CO_2_.

### Treatment with 5-aza-2′-deoxycytidine, trichostatin A, MGCD0103 or MC1568

Cell lines were treated with 1 μM 5-aza-2′-deoxycytidine (DAC, Sigma-Aldrich, USA) for 48 h, 0.3 μM trichostatin A (TSA, Sigma-Aldrich, USA) for 24 h, 20 μM MGCD0103 (Selleck, USA) for 24 h, or 20 μM MC1568 (Selleck, USA) for 24 h. Cell lines treated with dimethyl sulfoxide (DMSO, Sigma-Aldrich, USA) were used as controls.

### RNA extraction

Total RNA was extracted with TRIzol^@^ reagent (Invitrogen, USA) according to the manufacturer's instructions. RNAase-free DNAase I (Takara, Japan) was used to remove possible genomic DNA contamination. The concentration and quality of the total RNA were assessed with Nanodrop Spectrophotometer (NanoDrop 2000c, Thermo Fisher Scientific, USA).

### Semi-quantitative and real-time PCR

Reverse transcription was performed in 10 μl reaction volume using Reverse transcriptase M-MLV (RNase H-) (Takara, Japan) with a total of 1 μg of RNA. The abundance of gene expression was detected by relative quantitative real-time PCR with 7900 HT Real-Time PCR System (Applied Biosystems, USA) and SYBR green dye (Takara, Japan) according to the manufacturer's protocols. The 2^−ΔΔct^ calculation method was used to analyze the relative expression levels [46]. The primers that were used for *SEMA3E* amplification were as follows: forward, 5′-TAAAACTCAGACCCTCCTTAAAGT-3′, reverse, 5′-TGATGTTCTGTTCAGATTCAAGAGC-3′; primers for *P300*: forward, 5′-CCAAGCGGCCTAAACTCTCA-3′, reverse, 5′-GGTAAGTCGTGCTCCAAGTCA-3′; primers for housekeeping gene *GAPDH*, which was used as endogenous control: forward, 5′-CTCTGCTCCTCCTGTTCGAC-3′, reverse, 5′- GCGCCCAATACGACCAAATC-3′.

### Vector construction

The full length *SEMA3E* transcript variant 1 ORF (2328 bp, GenBank accession number NM_012431.2) was amplified from HGC-27 cDNA. The PCR product obtained was inserted into pCMV6-AC-RFP and pCMV6-AC-GFP (OriGene Technologies, USA) between the Sgf I and Mlu I sites. The primers used to amplify full length *SEMA3E* were as follows: forward, 5′-ATGCGATCGCATGGCATCCGCGGGGCACAT-3′, reverse, 5′-ATACGCGTTGCATGTGTGCCTGTTGGGT-3′. The accuracy of the insert sequence was confirmed by sequencing.

### Cell transfection

Cell transfection was performed with Lipofectamine^TM^ 2000 Transfection Reagent (Invitrogen, USA) according to the manufacturer's protocols.

### Luciferase reporter assay

The luciferase reporter assay was performed with Luciferase Assay System (Promega, USA) according to the manufacturer's instruction; luciferase activity was detected with Glomax 20/20 luminometer (Promega, USA). A 1376-bp sequence from −1655 bp to −280 bp upstream of *SEMA3E* transcription initiation site was cloned into PGL3-basic vector (Promega, USA) between the Xho I and Hind III sites upstream of the firefly luciferase gene. The recombinant plasmid was confirmed by sequencing.

### Cell viability and adhesion-dependent colony formation assay

Transiently transfected gastric cancer cells were seeded in 96-well plate at 1500–3000 cells per well for 6 days, and cell viability was detected with the Cell counting Kit-8 (Dojindo Laboratories, Japan). The optical density at 450 nm was measured to indicate cell viability at 24-h intervals.

Transfected gastric cancer cells were plated in 60-mm dishes at a density of 0.5-1 × 10^4^ cells per well for adhesion-dependent colony formation assay. G418 (Life technology, USA) was added to the culture medium at a final concentration of 0.6-1.2 mg/mL. Culture medium that contained G418 was changed every 3–4 days. Then, 3-4 weeks later, the remaining colonies were fixed with 4% paraformaldehyde and dyed with crystal violet. The colonies were counted according to the defined colony size.

### Cell cycle and cell apoptosis analysis

Forty eight hours after transfection, the cells were harvested and cell cycle distribution analysis was performed with FC500 Flow Cytometer (Beckman Coulter, USA) and DNAprep stain Kit (Beckman Coulter, USA) according to the manufacturer's instruction; data were analyzed by MultiCycle software.

Forty eight hours after transfection, the cells were collected and cell apoptosis analysis was performed in BD FACS Canto II Flow cytometry System (BD Biosciences, USA) with Annexin V : PE Apoptosis Detection Kit (BD Biosciences, USA) according to the manufacturer's protocol; all data were analyzed by FlowJo7.6.1 software.

### *In vitro* cell migration and invasion assay

Cell migration assay was performed on 24-well transwell inserts with 8-μm sized pores (BD Biosciences, USA). Cell invasion assay was performed onmatrigel invasion chamber with 8-μm sized pores (BD Biosciences, USA). Gastric cancer cells were trypsinized and washed three times in DMEM with 1% FBS. In all, 1 × 10^5^ cells were suspended in 500 μl DMEM with 1% FBS and added to the upper chamber, while 750 μl DMEM with 10% FBS and 10 μg/ml fibronectin (BD Biosciences, USA) was placed in the lower chamber. For the control, DMEM with 1% FBS was placed in the lower chamber. After 48 h of incubation, cells remained in the upper chamber were removed with flat-bottomed cotton swabs. Cells on the lower surface of the chamber were fixed with 4% paraformaldehyde and stained with 0.5% crystal violet. At least 6 random microscopic fields (magnification, × 200) were photographed, and the cells were counted.

### *In vivo* tumorigenicity

A suspension of MGC-803 cells (2 × 10^6^ in 200 μl sterile 1 × PBS) was inoculated into the right armpit of 5–6-week-old male BALB/c nude mice (Shanghai Experimental Animal Center, China). The tumor formation kinetics was estimated by measuring the tumor size at 3–4-day intervals. Digital caliper was used to measure tumor size, and tumor volume was calculated with the following formula: volume = 0.5 × width^2^ × length. All animal procedures were carried out with the approval of the Institutional Committee of Shanghai Jiao Tong University School of Medicine for Animal Research.

### Western blot

Forty eight hours after transfection, cell lysates were collected and separated on an 8–10% gel by SDS-PAGE and then transferred to a 0.2-μm PVDF membrane (Bio-Rad, USA). After blocking with Odyssey Blocking Buffer (Li-COR Biosciences, USA), the membrane was incubated with primary antibody (1:1000) at 4°C overnight, followed by incubation with IRDye 800CW or 680 secondary antibodies (1:5000, LI-COR Biosciences, USA). β-actin was used as endogenous control. The Odyssey Infrared Imaging System was used to visualize targeted protein bands.

### Immunofluorescence assay

Alexa Fluor 488 Phalloidin (Invitrogen, USA) was used to analyze F-actin content in gastric cancer cells according to the manufacturer's protocol. The stained cells were observed by Leica TCS SP5 confocal microscope (Leica microsystems, Germany).

### Statistical analysis

All statistically significant differences were evaluated by Student's *t*-tests, conducted with GraphPad Prism software and displayed as means ± standard deviation (SD), unless otherwise stated. *P* < 0.05 was considered to be significant.

## SUPPLEMENTARY FIGURES


