# Supplementary material for: Epigenetically downregulated Semaphorin 3E contributes to gastric cancer
Source: Oncotarget. 2015 May 12;6(24):20449–65. doi: 10.18632/oncotarget.3936 (PMC4653017; doi:10.18632/oncotarget.3936)
Supplement: Supplementary file 1 [file oncotarget-06-20449-s001.pdf]

SUPPLEMENTARY FIGURES

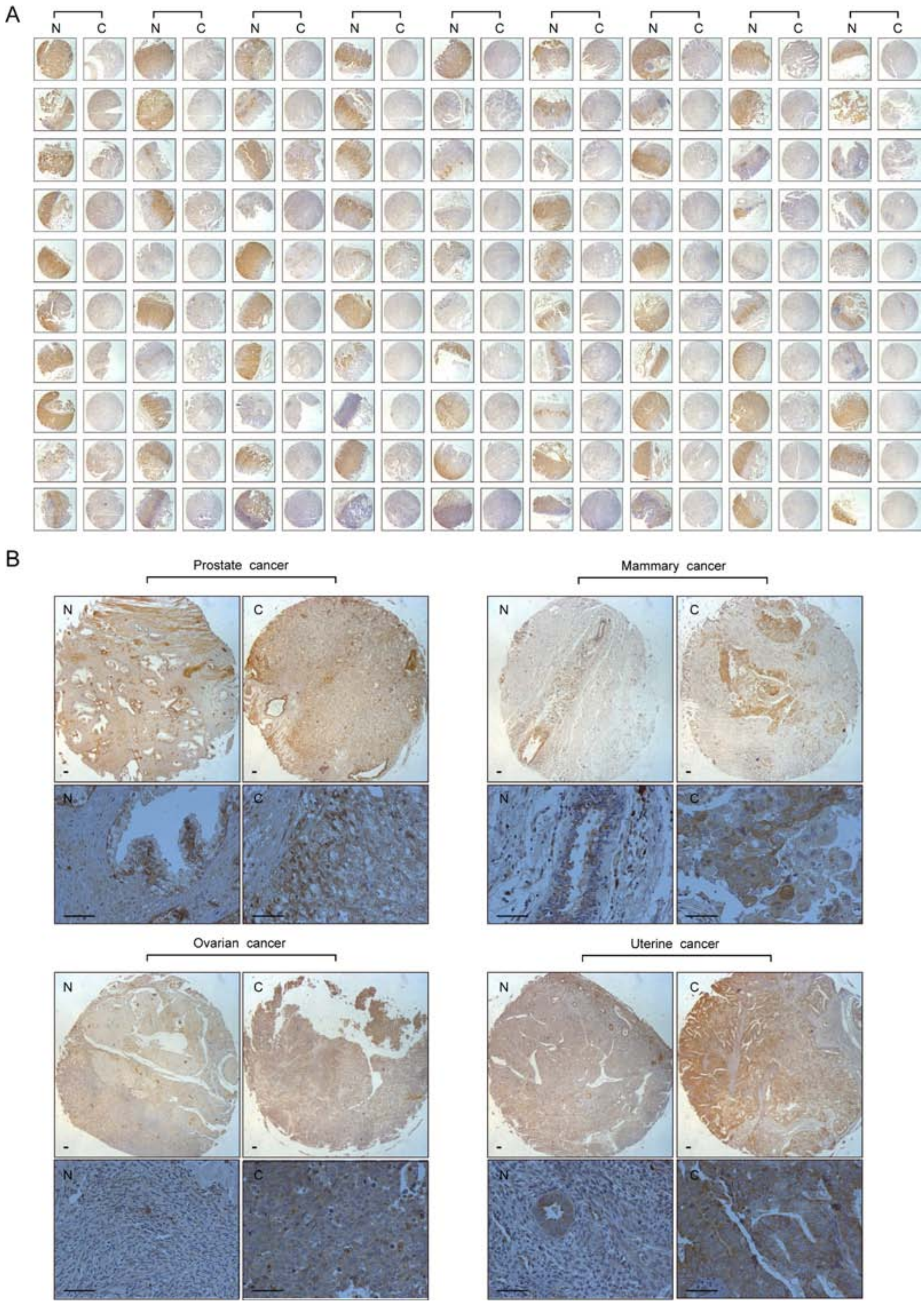

Supplementary Figure S1: Sema3E was down-regulated in gastric cancer and up-regulated in some other cancers. A. Sema3E expression was assessed by immunohistochemistry in 90 pairs of gastric cancer tissues and corresponding adjacent nontumor tissues ranged from TNM I to TNM IV. **B.** The expression of Sema3E in prostate cancer, mammary cancer, ovarian cancer and uterine cancer and corresponding adjacent normal tissues was analyzed by immunohistochemistry. Scale bar is 50 μm.

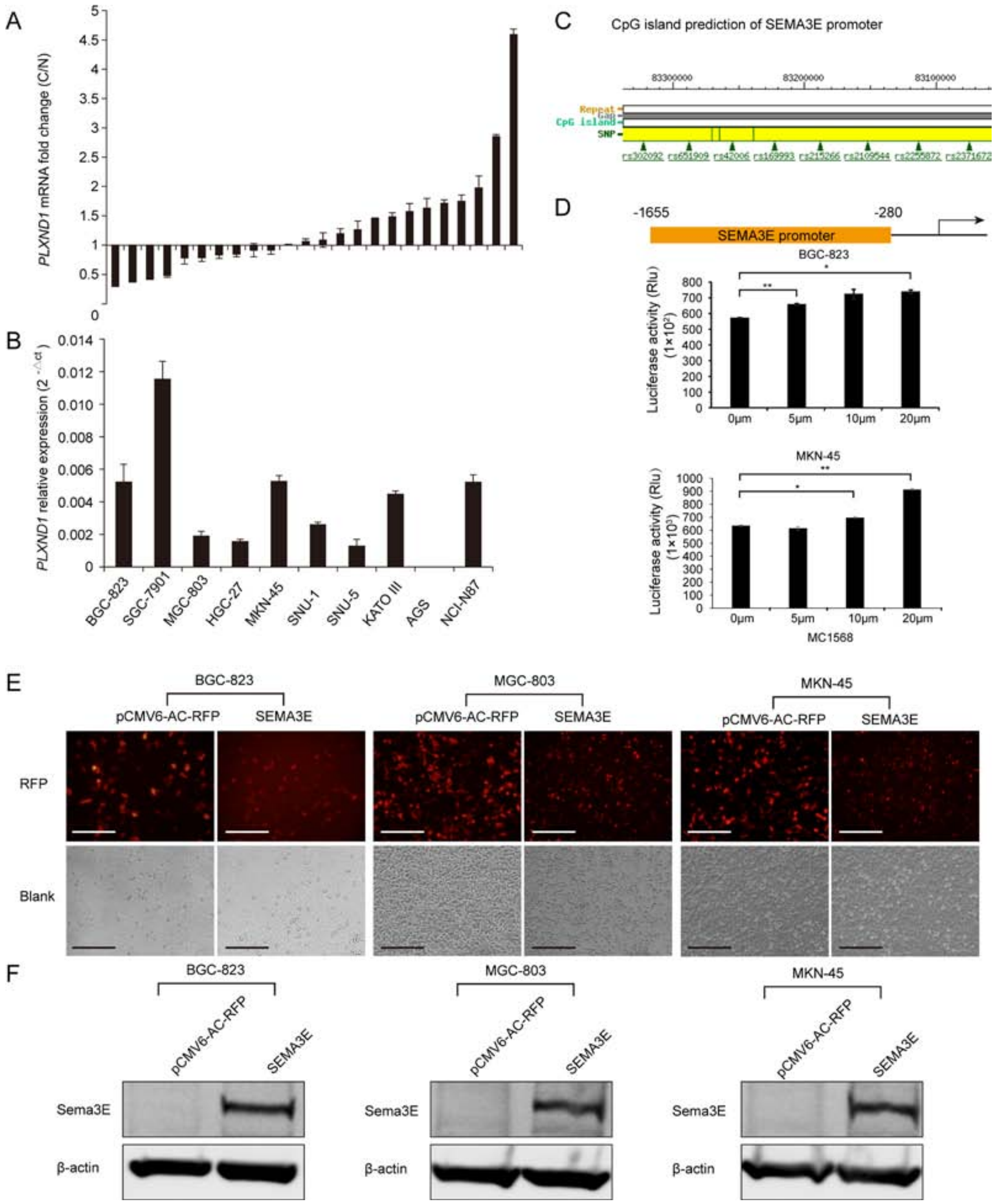

Supplementary Figure S2: The expression of *PLXND1* mRNA in gastric cancer and gastric cancer cell lines, CpG island prediction of *SEMA3E* promoter, effect of MC1568 on *SEMA3E* promoter activity, and the effect of ectopic expression of Sema3E in gastric cancer cells. A. The expression level of *PLXND1* mRNA in 24 pairs of gastric cancer and corresponding adjacent normal tissues. Level of *PLXND1* was determined by real-time PCR and normalized to the endogenous control (*GAPDH*). **B.** Real-time PCR was performed to analyze the expression level of *PLXND1* in gastric cancer cell lines. **C.** No CpG island was found on the promoter of *SEMA3E* according to the online prediction software DBTSS. **D.** Luciferase activity of *SEMA3E* promoter in BGC-823 and MKN-45 cells treated with MC1568 was analyzed. DMSO treatment was used as control. The data are presented as the mean ± SD. $^{**}P < 0.01$, $^{*}P < 0.05$ versus the control. **E.** The representative fields of forced Sema3E expression in gastric cancer cell lines by fluorescence microscopy are shown. Scale bar is 500 μm. **F.** Immunoblotting shows the ectopic expression of Sema3E with pCMV6-AC-RFP vector in these gastric cancer cell lines.

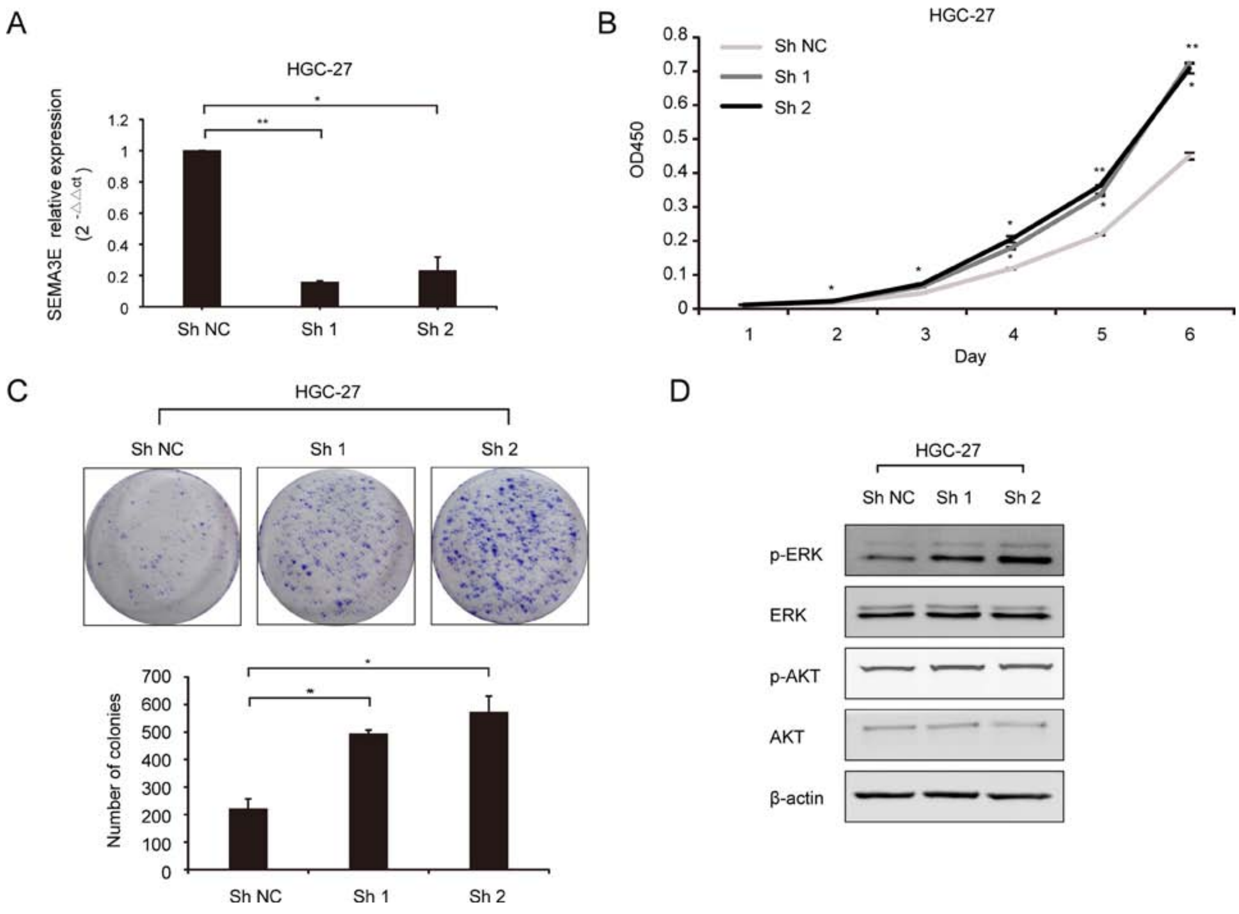

Supplementary Figure S3: Knockdown of Sema3E promoted gastric cancer cells proliferation and induced ERK1/2 phosphorylation. **A.** The effect of Sema3E knockdown in HGC-27 was shown. **B.** Knockdown of Sema3E expression promoted the proliferation of HGC-27 gastric cancer cell line. **C.** Sema3E knockdown promoted colony formation of HGC-27. Representative colony formation pictures of cells transfected with Sema3E ShRNA1, ShRNA2 and control vectors are shown. The histograms represent the number of colonies, and the data are shown as the mean ± SD. **D.** Knockdown of Sema3E increased ERK1/2 phosphorylation in HGC-27. The experiments were all repeated at least 3 times to confirm the reproducibility of the results. $^{**}P < 0.01$, $^{*}P < 0.05$ versus the control.

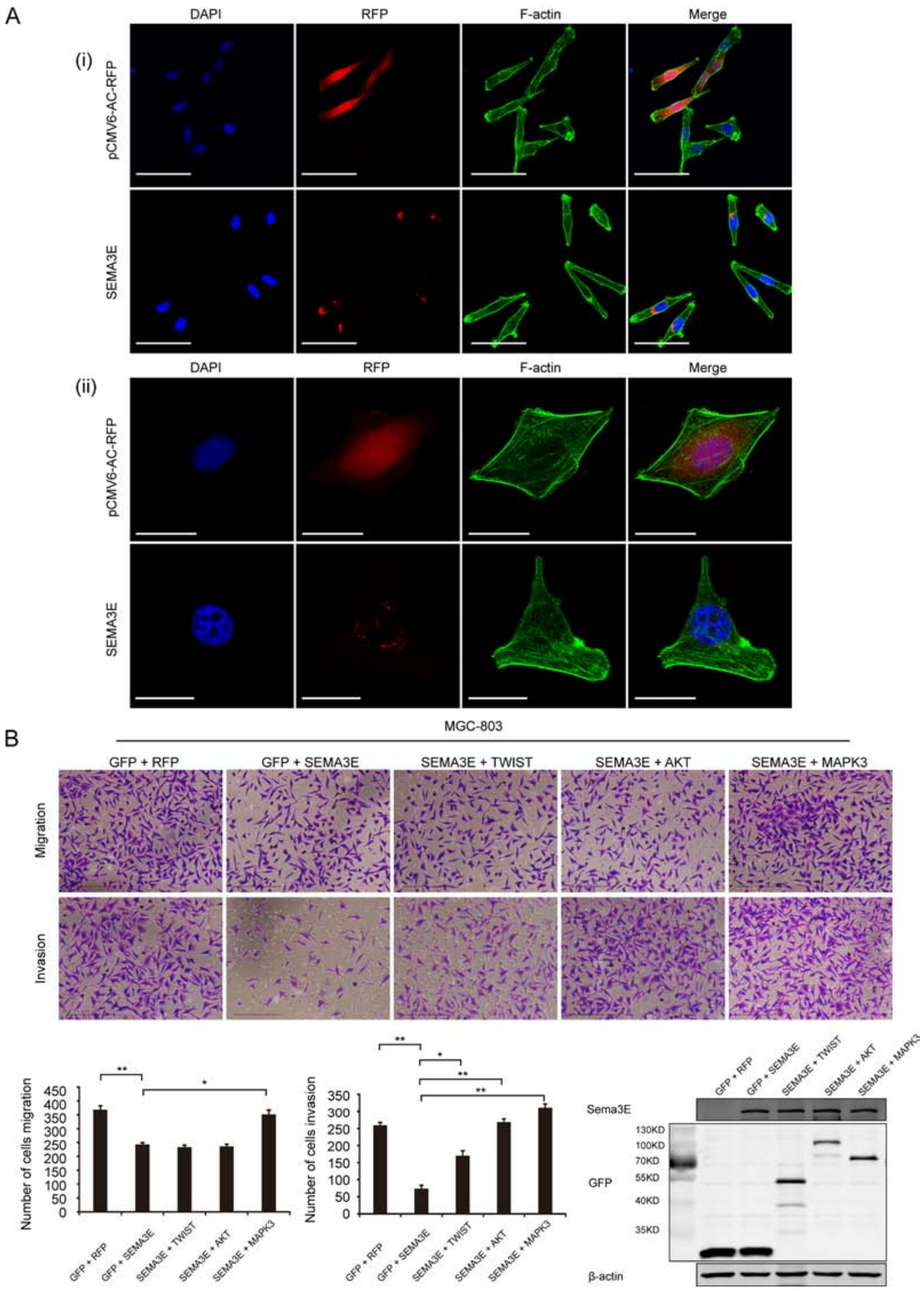

Supplementary Figure S4: Sema3E overexpression had no effect on F-actin content. Rescue assay was performed to find whether Twist, Akt and ERK1 can abolish Sema3E's inhibitory effect on migration and invasion of gastric cancer cells. **A.** Phalloidin staining was performed to detect F-actin content in MGC-803 cells after Sema3E overexpression. Scale bar is 20 μm. **B.** Overexpression of MAPK3 can abolish Sema3E's inhibitory effect on MGC-803 cells migration. And overexpression of Twist, Akt and MAPK3 can abolish Sema3E's inhibitory effect on MGC-803 cells invasion. All experiments were repeated at least three times. Representative fields of cells that invaded are shown. The histogram indicates the number of cells that invaded; the data are shown as the mean ± SD. Scale bar is 500 μm**.**

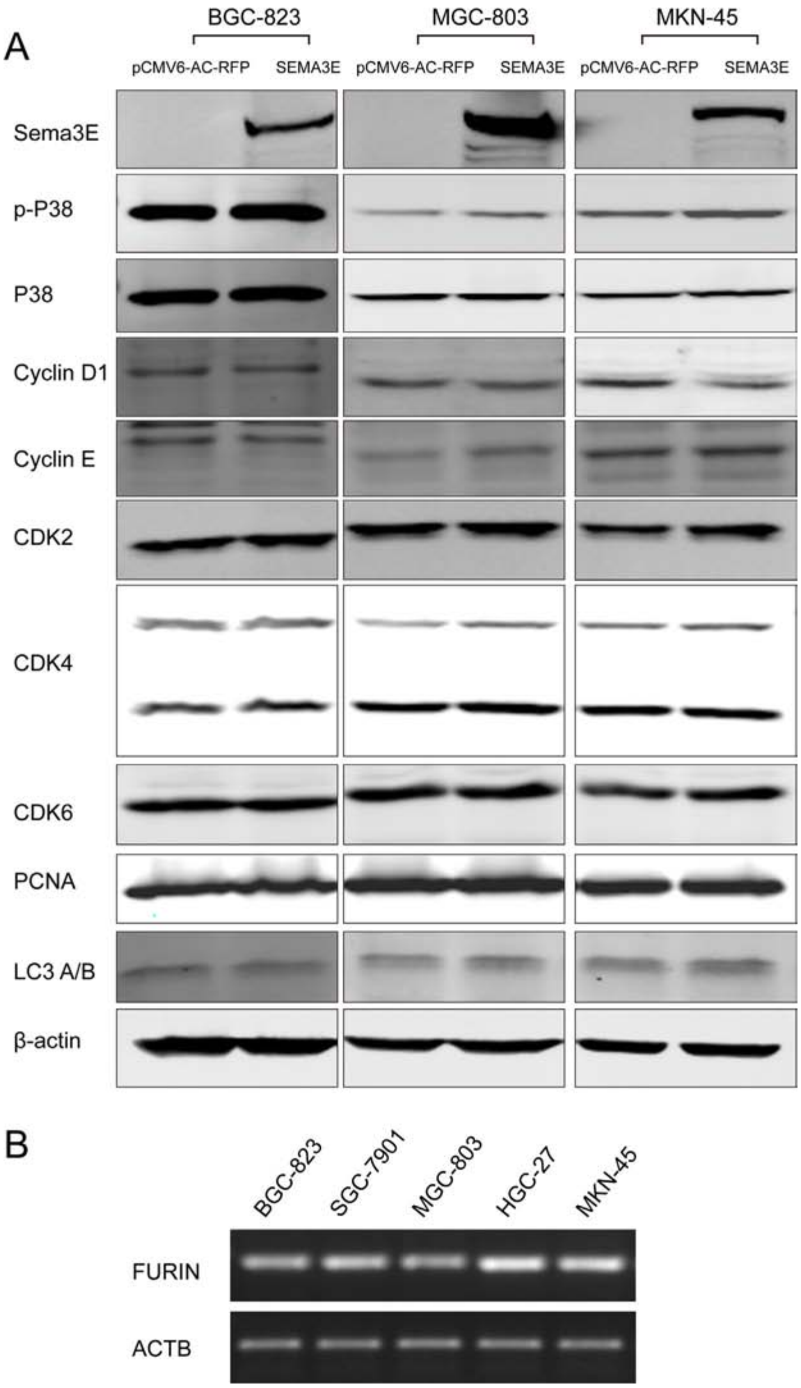

Supplementary Figure S5: A. Screening for important cell signaling pathway nodes involved in inhibitory effect of Sema3E on gastric cancer cells. B. RT-PCR indicates the expression of *FURIN* in gastric cancer cells**.**
